# Enhanced uptake, high selective and microtubule disrupting activity of carbohydrate fused pyrano-pyranones derived from natural coumarins attributes to its anti-malarial potential

**DOI:** 10.1186/s12936-019-2971-z

**Published:** 2019-10-11

**Authors:** Sonal Gupta, Juveria Khan, Priti Kumari, Chintam Narayana, R. Ayana, Malabika Chakrabarti, Ram Sagar, Shailja Singh

**Affiliations:** 1 Special Centre for Molecular Medicine, Jawaharlal Nehru University, New Delhi, 110067 India; 2 School of Biotechnology, Jawaharlal Nehru University, New Delhi, 110067 India; 3 Department of Chemistry, Shiv Nadar University, NH-91 Dadri, GB Nagar, Greater Noida, UP 201314 India; 4 Department of Life Sciences, School of Natural Sciences, Shiv Nadar University, Greater Noida, India; 5 Department of Chemistry, Institute of Science, Banaras Hindu University, Varanasi, 221005 India

**Keywords:** *Plasmodium falciparum* 3D7, Carbohybrids, Carbohydrate-fused pyranopyrone, Microtubule, Coumarins, Malaria

## Abstract

**Background:**

Malaria is one of the deadliest infectious diseases caused by protozoan parasite of *Plasmodium* spp. Increasing resistance to anti-malarials has become global threat in control of the disease and demands for novel anti-malarial interventions. Naturally-occurring coumarins, which belong to a class of benzo-α-pyrones, found in higher plants and some essential oils, exhibit therapeutic potential against various diseases. However, their limited uptake and non-specificity has restricted their wide spread use as potential drug candidates.

**Methods:**

Two series of carbohydrate fused pyrano[3,2-c]pyranone carbohybrids which were synthesized by combination of 2-*C*-formyl galactal and 2-*C*-formyl glucal, with various freshly prepared 4-hydroxycoumarins were screened against *Plasmodium falciparum*. The anti-malarial activity of these carbohybrids was determined by growth inhibition assay on *P. falciparum* 3D7 strain using SYBR green based fluorescence assay. Haemolytic activity of carbohybrid **12**, which showed maximal anti-malarial activity, was determined by haemocompatibility assay. The uptake of the carbohybrid **12** by parasitized erythrocytes was determined using confocal microscopy. Growth progression assays were performed to determine the stage specific effect of carbohybrid **12** treatment on Pf3D7. In silico studies were conducted to explore the mechanism of action of carbohybrid **12** on parasite microtubule dynamics. These findings were further validated by immunofluorescence assay and drug combination assay.

**Results:**

2-C-formyl galactal fused pyrano[3,2-c]pyranone carbohybrid **12** exhibited maximum growth inhibitory potential against *Plasmodium* with IC_50_ value of 5.861 µM and no toxicity on HepG2 cells as well as no haemolysis of erythrocytes. An enhanced uptake of this carbohybrid compound was observed by parasitized erythrocytes as compared to uninfected erythrocytes. Further study revealed that carbohybrid **12** arrests the growth of parasite at trophozoite and schizonts stage during course of progression through asexual blood stages. Mechanistically, it was shown that the carbohybrid **12** binds to α,β-heterodimer of tubulin and affects microtubule dynamics.

**Conclusion:**

These findings show carbohydrate group fusion to 4-hydroxycoumarin precursor resulted in pyrano-pyranones derivatives with better solubility, enhanced uptake and improved selectivity. This data confirms that, carbohydrate fused pyrano[3,2-c]pyranones carbohybrids are effective candidates for anti-malarial interventions against *P. falciparum*.

## Background

Malaria is a life-threatening mosquito-borne disease caused by apicomplexan parasite of genus *Plasmodium*. Progress in the global malaria control has stalled due to various factors including unavailability of vaccine and intensification of drug resistant malaria. Clinical and parasitological outcomes observed during routine therapeutic efficacy studies of artemisinin-based combination therapy have raised concern over the emergence and possible spread of parasitic resistance at a wider scale. Thus, development of new effective drugs is the desperate need of the hour.

Coumarins belonging to the benzo-α-pyrones class of natural products have drawn significant interest owing to their therapeutic properties [1]. Coumarins and their synthetic derivatives have shown to be effective antimicrobial and anticancer agents [2, 3]. Furthermore, different derivatives of coumarin have been shown to inhibit proliferation via targeting microtubule dynamics [4, 5]. Tubulin is identified as one of the potential targets of coumarin derivatives. Tubulin inhibitors hamper the proper growth and multiplication of malaria parasite in vitro and in vivo [6, 7].

In the present work, thirty novel pyrano[3,2-c]pyranone derivatives which were prepared by the combination of 2-*C*-formyl galactal and 2-*C*-formyl glucal with various freshly prepared 4-hydroxycoumarins as precursor compounds under microwave assisted condition as carbohydrate fused hybrids were screened against *Plasmodium falciparum*. The aim behind this hybridization was to improve their bioavailability and selectivity. Selected members from this library were found to possess anticancer activity [8]. Anti-malarial potential of these compounds was evaluated against *P. falciparum* 3D7 strain by growth inhibition assay. 2-*C*-formyl galactal fused pyrano[3,2-c]pyranone carbohybrid **12** exhibited maximum inhibitory potential selectively against *P. falciparum* 3D7. Parasite death has been evaluated by measurement of mitochondrial membrane potential using the live-dead cell staining fluorometric dyes 5,5′6,6′-tetrachloro-1,1′,3,3′ tetraethylbenzimidazolyl carbocyanine iodide (JC-1). Different derivatives of coumarin have been shown to inhibit cell proliferation via targeting microtubule dynamics in eukaryotes [9]. Carbohybrid **12** has shown inhibitory effects on mammalian tubulin as reported in the previous study [8]. This led to the hypothesis that *P. falciparum* growth inhibition induced by carbohybrid **12** is accredited to its anti-microtubule activity in the parasite. Therefore, the effect of carbohybrid **12** on parasite microtubule dynamics was investigated through immunofluorescence assays and in silico studies. This study indicates anti-malarial potential of carbohydrate fused pyrano[3,2-c]pyranone carbohybrid **12** and mechanistic insights into its inhibitory activity.

## Methods

### Chemistry

The privileged carbohydrate-fused pyrano[3,2-c]pyranone, carbohybrid **12** and other compounds were prepared using freshly synthesized 4-hydroxycoumarins reacting it with 2-*C*-formyl galactal and 2-*C*-formyl glucal under microwave assisted reaction conditions. The structure of carbohydrate-fused pyrano[3,2-c]pyranone, carbohybrid **12** was established by 1D and 2D NMR (NOE, COSY and HSQC) experiments and comparing it with earlier spectral data. The details of the synthetic design and reaction optimization for the synthesis of these compounds can be obtained from the previous study [8].

### Reagents and antibodies

RPMI 1640 medium, Albumax II and gentamicin sulphate (Invitrogen, USA), sodium bicarbonate, sorbitol, methanol, giemsa, SYBR green, glutaraldehyde, 2-deoxy-2-[(7-nitro-2,1,3-benzoxadiazol-4-yl)amino]-d-glucose (2-NBDG), hypoxanthine, paclitaxel, colchicine, bovine serum albumin [Sigma-Aldrich, (USA)], Alexa-fluor 546 conjugated anti-mouse IgG goat sera, ProLong Gold antifade reagent with DAPI (4′,6-diamidino-2-phenylindole) (Life technologies-Invitrogen, USA).

### Parasite culture

Blood stages of *P. falciparum* 3D7 strain and RKL9 strains were cultured using a standard protocol [10]. Briefly, parasites were grown in complete RPMI 1640 medium (RPMI 1640 medium with 2 mM l-glutamine, 25 mM HEPES, 2 g L^−1^ NaHCO_3_, 27.2 mg L^−1^ hypoxanthine and 0.5% Albumax II, pH 7.4) using O^+^ human RBC. The culture was maintained at 2–4% haematocrit and incubated at 37 °C in a mixed gas (5% CO_2_, 5% O_2_ and 90% N2) chamber. The parasites were tightly synchronized by sorbitol treatment for progression assays and 96 h growth assessment assay. Parasitaemia was monitored by making thin blood smears, fixed with methanol, and stained with 10% Giemsa for 10 min and then observed under light microscope at 100×. The parasitaemia was monitored by counting the number of parasitized cells in estimated 2000–4000 erythrocytes.

### SYBR green based fluorescence assay

In vitro anti-plasmodial activities of synthesized novel pyrano[3,2-c]pyranone derivatives were determined using SYBR green based fluorescence assay [11, 12]. Briefly, the synchronized parasite cultures were diluted at ring stage at an initial parasitaemia of 0.8% and 2% haematocrit and treated with different compounds at 10 μM concentration till trophozoite stage i.e. approx. 60 h post treatment. 50 mM stock solution of these compounds was prepared in 100% dimethyl sulfoxide (DMSO) and stored at − 20 °C. The assay plates were frozen at − 80 °C to stop growth and lyse erythrocytes. 100 µL of lysis buffer containing SYBR green (0.2 µL of SYBR green I/mL of lysis buffer) was added to each well and incubated for 3 h in the dark at 37 °C. The fluorescence intensity was determined at 485 nm excitation and 530 nm emission using a Varioskan Flash multi-well plate reader (Thermo Scientific). The data was corrected for the background fluorescence of uninfected erythrocytes, normalized to the growth of control parasites. The percent inhibition was calculated with respect to untreated control. IC_50_ and IC_90_ values of carbohybrid **12** were determined by plotting values of percent inhibition against log concentration of compound varying from 1 to 50 µM using Graphpad PRISM software.

### Light microscopy

Parasitized erythrocytes culture, control and carbohybrid **12** treated were taken at different time points of asexual blood stages and thin blood smears were made on glass slides. Slides were fixed in methanol, air dried and stained with Giemsa for examination at 100× under light microscope. The images were captured using CatCam camera and processed by Catymage software (Catalyst Biotech).

### Atomic force microscopy (AFM)

Thin smears of the parasite cultures, untreated control or carbohybrid **12** treated were prepared for AFM imaging at the different stages of the infected erythrocytes. WITec alpha 300RA was used to image both the smeared samples. NSG30 probes with force constant of 22–100 N/m, resonant frequency of 240–440 Hz, Tip curvature radius of 10 nm (Tips nano) were used to image the smeared cells in non contact mode. Topographic images were obtained at points per line and lines per image of 256 * 256 with the scan rate of 0.5 times/line (Trace) (s). All the AFM images were recorded using the Control Four 4.1 software. The images were processed and analysed using software Project Four 4.1 software (WITec).

### Haemocompatibility assay

To assess the effect of carbohybrid **12** on host erythrocytes, haemolysis assays were conducted by standard protocols [13]. Briefly, the erythrocytes suspension was prepared at 50% haematocrit in PBS. 50 µL of this suspension was pre-incubated with different concentrations of carbohybrid **12** in 1.5 mL 1× PBS. 50 µL of cell suspension with 1.5 mL PBS was taken as the negative control for haemolysis. For positive control, 50 µL of cell suspension was added to 1.5 mL of deionized water (hypotonic for erythrocytes). PBS with various concentrations of carbohybrid **12** was taken as blank for each test sample. After incubation at 37 °C for an hour, the reaction was stopped by adding 50 µL of 2.5% glutaraldehyde. Pellet was removed after centrifugation at 1000×*g* at 4 °C for 5 min and the absorbance of the supernatant was measured at 415 nm. The experiment was performed in duplicates. The percent haemolysis was calculated as: (Absorbance of sample/Absorbance of positive control) × 100%.

### Uptake assay

Parasites at trophozoite stage were treated with 6 µM of carbohybrid **12** and its precursor, compound **5**. The cells were observed for autofluorescence at different time intervals from 30 min to 2 h in UV filter using confocal microscope (Olympus 1000). In addition, the cells treated for 2 h were washed with incomplete RPMI and incubated at 37 °C with 200 µM of NBD-G for 30 min in dark. The fluorescence was observed at 100× using confocal microscope (Olympus 1000).

### JC-1 staining for estimation of mitochondrial membrane potential

JC-1 exists as monomers in cytoplasm and emits green fluorescence (525 nm) and in mitochondrion, it forms aggregates emitting red fluorescence (590 nm). To estimate any changes in mitochondrial membrane potential as a marker for cell death, parasite culture treated with carbohybrid **12** was incubated with 5 μM JC-1 for 30 min at 37 °C, washed twice with 1× PBS and imaged using confocal laser scanning microscope (Olympus1000). The fluorescence intensity for 30 cells in each channel was calculated using FV10ASW 1.7 Viewer software. The ratio of red to green fluorescence for each cell was calculated and plotted in the bar graph using MS Excel software.

### Progression assay

The effect of carbohybrid **12** was tested on development of *P. falciparum* 3D7 strain through different blood stages i.e. rings, trophozoites and schizonts. Ring-stage parasite culture was diluted to 1% parasitaemia and 2% haematocrit in complete RPMI medium. The culture was treated with carbohybrid **12** at two different concentrations i.e. 8 µM and 15 µM and incubated at 37 °C for 48 h. To determine the effect of carbohybrid **12** in stage-specific manner, parasite culture was also treated at trophozoite and schizonts stages and observed till ring stage formation in the next cycle. The parasitaemia at each stage was calculated by counting 5000 cells in Giemsa stained smears under 100× objective by light microscope.

### Combination assay

Synchronized ring stage parasites with 0.5% parasitaemia and 2% haematocrit were seeded in a 96 well plate. The culture was treated with carbohybrid **12** (1.25 µM to 20 µM) and paclitaxel (25 nm to 400 nm) at various concentrations according to fixed ratio method. In addition, both the drugs were individually tested. The plate was incubated at 37 °C for 96 h. The media was changed with appropriate drug concentrations after every 24 h. Untreated culture was negative control for the experiments. The percent parasitaemia with respect to untreated control was determined using SYBR green dye.

### Molecular docking

The crystal structure of *P. falciparum* tubulin has not yet been determined, so individual monomers of parasite alpha and beta-tubulin were modelled based on sequence homology modelling using SWISS-MODEL server [14]. The models were tested for quality using RAMPAGE Ramachandran plotting program. The dimeric form of *Plasmodium* tubulin was constructed by assembling two modelled monomers using the protein–protein docking server ClusPro [15]. The 3D structure of the paclitaxel was downloaded from PUBCHEM database. The PUBCHEM structure which was in SDF format was converted to PDB using the Open Babel program [16]. The 3-D structure of the carbohybrid **12** was constructed using Marvinsketch sketch module. Molecular docking of the protein dimer with paclitaxel and carbohybrid **12** was performed using AUTODOCK [17]. Clusters were generated with an RMS tolerance of 2 Å. A docking pocket of volume 40 Å was used for molecular docking of these small molecules. Docked poses were rendered with PyMOL.

### Confocal microscopy

The tubulin staining in *P. falciparum* untreated and carbohybrid **12** treated cultures was monitored by immunofluorescence assay. Briefly, ring and trophozoite stage parasites were treated with 6 µM (IC_50_) carbohybrid **12** and incubated for 24 h. Parasites were treated with modulators of microtubule dynamics namely 10 µM colchicine and 500 nM paclitaxel [13]. Thin smears of *P. falciparum* infected erythrocytes at schizont stages were fixed in chilled methanol and air dried. Fixed slides were incubated in the blocking buffer containing 5% BSA in 1× PBS for 1 h at room temperature. *P. falciparum* alpha-tubulin recombinant protein was produced in *Escherichia coli*. Primary antibody i.e. alpha tubulin raised against recombinant protein (1:200 dilution) was added to the blot and incubated for 1 h at room temperature. The slides were washed two times with PBS-T (1× PBS with 0.05% Tween-20) followed by 1× PBS washes and incubated with Alexa fluor 546 conjugated antimouse IgG goat sera at a dilution of 1:500. Finally, the slides were mounted with ProLong Gold antifade reagent with DAPI (4′,6-diamidino-2-phenylindole). Slides were observed in confocal microscope (Olympus 1000) to investigate the localization of tubulin in parasite.

### Thermal shift assay (TSA)

*Plasmodium falciparum* alpha-tubulin recombinant protein was expressed in *E. coli* and purified by affinity chromatography. Purified alpha-tubulin protein and carbohybrid **12** interaction was monitored via thermal shift assay. 2.5 µg of protein was heated at different temperatures in presence and absence of 50 µM of carbohybrid **12** for 6 min and then cooled at room temperature for 3 min. Unheated protein in absence and presence of drug was taken as control. Following centrifugation at 14,000×*g* for 40 min at 4 °C, supernatant was transferred to new tubes and run on SDS. The change in the protein stability in the presence and absence of compound was monitored by running the sample on SDS-PAGE.

## Results

### Anti-malarial efficacy of carbohydrate fused pyrano-[3,2-c]pyranones

The anti-malarial activity of carbohydrate fused pyrano[3,2-c]pyranones **11**–**20** and **21a/b**–**30a/b** (n = 20) along with different derivatives of 4-hydroxycoumarin parent compounds **1**–**10** (n = 10) (Additional file 1: Figure S1) at 10 µM was determined by growth inhibition assay (GIA) in *P. falciparum* 3D7 strain. The results showed that most of carbohydrate fused compounds inhibited growth of parasite in vitro (Additional file 1: Table S1). These compounds exhibited no cytotoxicity even at higher concentration of 100 µM against HepG2 cells (liver hepatocellular carcinoma) as described in the previous work [8].

**Fig. 1:**
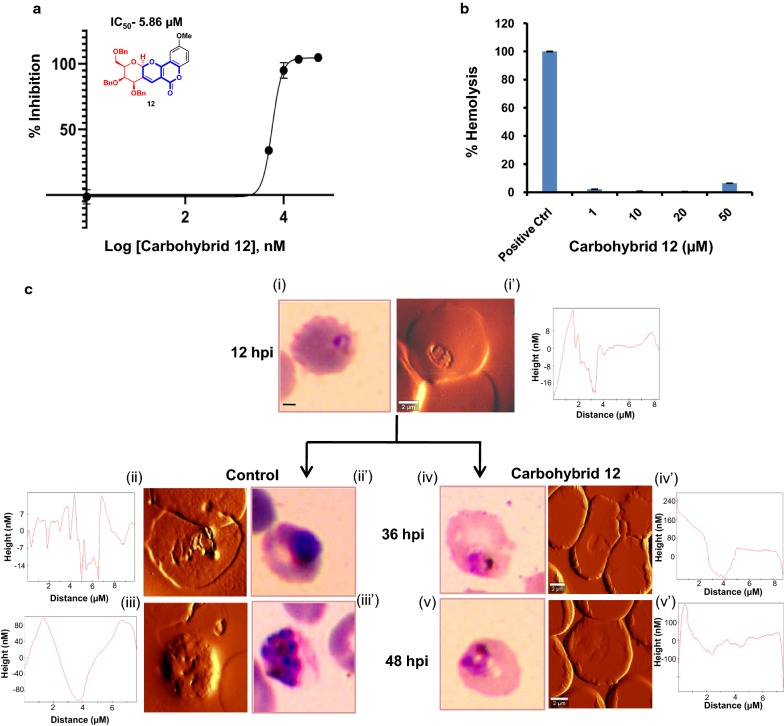
Cytotoxic effect of galactal fused pyrano-pyranone carbohybrid **12**. **a** To calculate half maximal inhibitory concentration (IC_50_) of carbohybrid **12**, *P. falciparum* 3D7 culture at ring stage was treated with different concentrations (1–50 µM) for one growth cycle. IC_50_ value was determined by plotting the values of percent growth inhibition against log concentrations of carbohybrid **12**. The experiments were performed in duplicates, n = 2 and ± SD value was calculated for each data point. **b** Percent haemolysis was calculated at different concentrations of carbohybrid **12**. Haemolysis was measured as percent of positive control. The released haemoglobin in the supernatant was detected by spectrophotometer at 415 nm. The average of two set of independent experiments for each data point ± standard error of the mean is shown. **c** Morphological changes in *P. falciparum* treated with carbohybrid **12** was observed by Giemsa staining (i)–(v) and Atomic force Microscopy (AFM) (i′)–(v′). Parasite cultures were treated with carbohybrid **12** at ring stages of 12 h post infection (hpi) as shown in (i, i′). Panel (ii), (ii′) and (iii), (iii′) represents morphology of trophozoites and late schizonts respectively in untreated control. Panel (iv), (iv′) and (v), (v′) shows the morphological changes in carbohybrid **12** treated parasites at trophozoite and schizonts stages respectively. Respective width/height plots for each stage is shown adjacent to their AFM images. Scale bar = 2 µm

Carbohybrid **12** exhibited potent anti-malarial effect against *P. falciparum* 3D7 strain with IC_50_ and IC_90_ values of 5.861 µM and 9.8 µM (Fig. 1a). All the tested concentrations of carbohybrid **12** produced a comparable growth inhibition against chloroquine resistance *P. falciparum* RKL-9 strain (Additional file 1: Figure S2). The IC_50_ value of carbohybrid **12** against *P. falciparum* RKL-9 strain was 3.563 µM. This indicates that this compound is efficient against both the chloroquine sensitive and the resistant strains. Upon structural analysis of this compound as depicted by structure activity relationship (SAR) studies, carbohybrid **12** has 2-OMe group at C-2 position which might be playing an important role for its anti-malarial activity [8]. To assess whether the inhibitory potential of carbohybrid **12** is not due to haemolytic effect of compound itself, uninfected erythrocytes were incubated with different concentrations of compound (1–50 μM). Carbohybrid **12** showed no significant haemolytic activity (< 5%) as compared to positive control i.e. deionized water (Fig. 1b).

### Carbohybrid 12 induces morphological changes in the parasite

Previous reports have suggested that treatment of *P. falciparum* with anti-malarial drugs lead to morphological alterations [18]. In order to determine the gross effects of carbohybrid **12** treatment on parasite morphology, treated cultures were compared with untreated control in time and stage specific manner. Figure 1c depicts morphological changes in *P. falciparum* cultures treated with 8 µM carbohybrid **12** as visualized by light microscopy at different points (12 h, 36 h and 48 h post infection). In comparison to untreated culture, shrinkage and aggregation of nuclear material of the parasites was observed in the late stage trophozoites of compound treated cultures. The parasite growth slowed down and the cytoplasm was found to be condensed. The effect was more evident in schizonts. Further using atomic force microscope (AFM) imaging the stage specific morphological alterations of *P. falciparum* infected erythrocyte in presence of carbohybrid **12** were analysed. Carbohybrid **12** treatment alters topography of parasitized erythrocyte. The reduction in parasitized erythrocyte membrane roughness was observed followed by carbohybrid treatment as compared to the untreated control (Fig. 1c, AFM width/height plots).

### Carbohybrid 12 enters efficiently in the parasitized erythrocytes

**Fig. 2:**
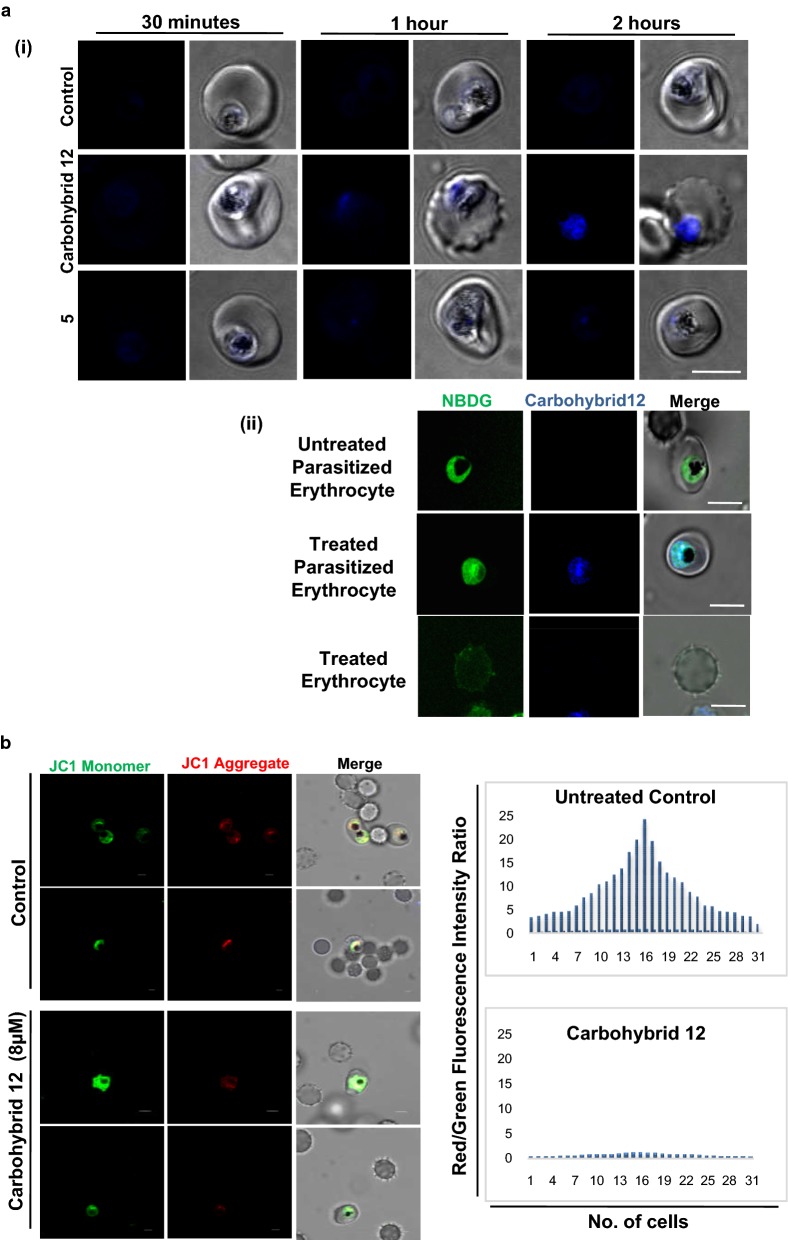
Uptake of carbohybrid **12** by *P. falciparum* induces cell death. **a** (i) Cellular uptake images of parasitized erythrocytes at different time points from 30 min to 2 h of incubation with carbohybrid **12** (6 µM) and precursor compound 5 (6 µM) (ii) carbohybrid **12** treated parasitized cells for 2 h were incubated with 2-NBDG for 30 min. Carbohybrid **12** shows increase in uptake in the time dependent manner. Uninfected erythrocytes treated with carbohybrid **12** showed less uptake of compound as compared to parasitized erythrocytes. **b** Fluorescence images of parasites showing JC-1 aggregates (red) in the mitochondria and monomeric JC-1 (green) in the cytoplasm. The first two rows of images shows untreated parasites with bright red signal at 590 nm indicating a functional mitochondrion; the last two rows show carbohybrid **12** treated dead parasites with bright green and very faint red signal. The bar graph showing the ratio of JC-1 (red)/JC-1 (green) in parasite population after treatment with carbohybrid **12** w.r.t control is shown in right panel. Scale bar = 5 µm

The conjugation of carbohydrate precursors like 2-C formyl galactal to pyrano[3,2-c]pyranone carbohybrid **12** was carried out with the aim to improve uptake of compound by infected cells. The intracellular uptake of carbohybrid **12** and its precursor, compound 5 was observed in infected parasite. These compounds show autofluorescence properties due to the presence of conjugated double bonds in the pyrano [3,2-c]pyranone ring. These results indicated higher cellular uptake of galactal-fused pyrano[3,2-c]pyranone carbohybrid **12** by parasitized erythrocytes as compared to its precursor. The cellular uptake of carbohybrid **12** was found to be gradually increasing with time from 30 min to 2 h (Fig. 2a(i)). Further, the uptake of fluorescent d-glucose analog i.e 2-[*N*-(7-nitrobenz-2-oxa-1,3-diazol-4-yl) amino]-2-deoxy-d-glucose (2-NBDG) was monitored in parasitized erythrocytes for measuring cellular uptake of glucose as compared to uninfected control cells (Fig. 2a(ii)). This could be linked to better uptake of carbohydrate conjugated carbohybrid **12** than its precursor 4-hydroxy coumarin in infected erythrocytes. This study suggests that the conjugation of carbohydrate moiety to pyrano[3,2-c]pyranone is improving uptake of compound by infected cells. These findings suggest that carbohybrid **12** might utilize glucose transporters to enter in the cells [19, 20].

### Effect of carbohybrid **12** on mitochondrial membrane potential as an indicator of cell death

The disruption of mitochondrial membrane potential (∆Ψ_m_) is a characteristic feature of cells undergoing apoptosis [21]. The change in membrane potential is determined by staining cells with mitoprobe dye JC1. In living cells, when ∆Ψ_m_ is normal, the dye aggregates inside the mitochondria and gives red fluorescence. However in case of membrane potential disruption, the dye remains in monomeric form in the cytoplasm and gives green staining. In this study, changes in ∆Ψ_m_ treated with carbohybrid **12** (8 µM) was monitored in parasitized erythrocyte. In the untreated control, the parasites showed bright red staining as a result of JC-1dye aggregation in the mitochondria, indicating the presence of viable cells (Fig. 2b). Confocal microscopy indicates increased green fluorescence in cytoplasm and reduced red staining in the mitochondria of the carbohybrid **12** treated parasites after 24 h of treatment. The ratio of red:green fluorescence in the control cells was higher as compared to carbohybrid **12** treated parasites as represented by the bar graphs. The results evidently confirmed the effect of carbohybrid **12** on mitochondria membrane potential (Δ*ψ*_m_) in the parasite which might lead to programmed cell death of parasite, however detailed studies need to be done.

### Carbohybrid **12** arrests growth at ring and trophozoite stages

**Fig. 3:**
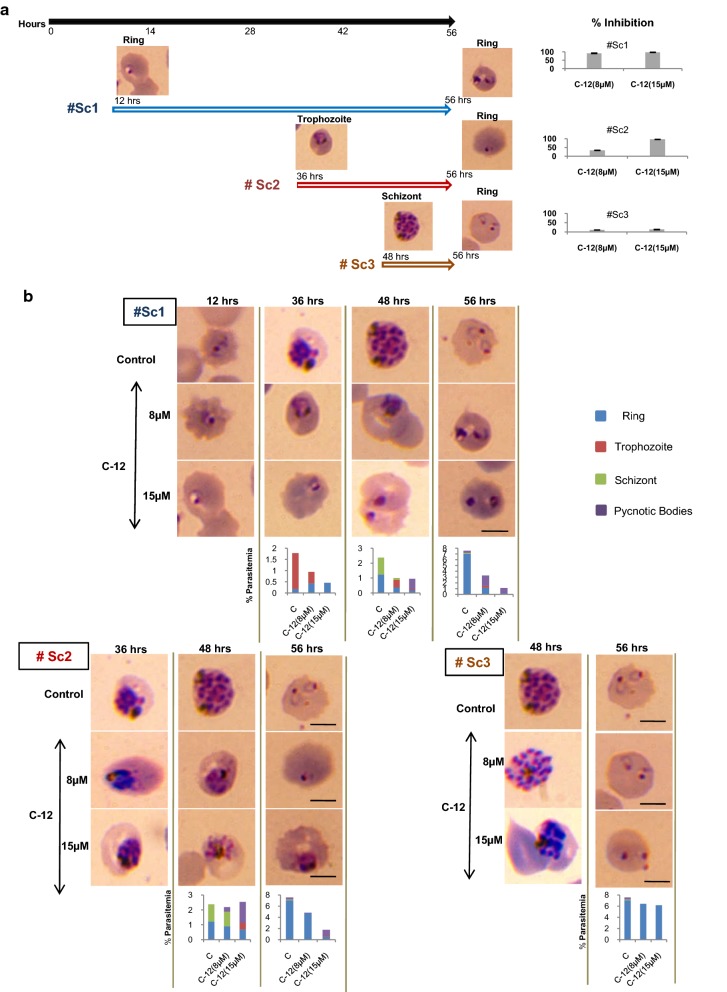
Effect of carbohybrid **12** on progression through asexual blood stages of *P. falciparum.* **a** Timeline representing different time points in the blood stage developmental cycle of *P. falciparum* (black line). # Sc1, # Sc2 and # Sc3 represent different time points of treatment with carbohybrid **12** at rings, trophozoites and schizonts respectively. The bar graph shows percent growth inhibition upon carbohybrid **12** treatment with respect to (w.r.t.) control. **b** *P. falciparum* 3D7 cultures were treated with carbohybrid **12** (C-**12**) at 8 µM or 15 µM at rings (upper panel, # Sc1), trophozoites (bottom left, # Sc2) or schizonts (bottom right, # Sc3). Morphology of *P. falciparum* was observed by Giemsa stained smears at different timelines of progression through blood stages in each set as shown. The bar graph in each column represents percent parasitaemia w.r.t. untreated control

During asexual stages of development in host erythrocytes, *Plasmodium* replicates mitotically and produces different stages like rings, trophozoite and schizonts in host erythrocytes. The late schizonts rupture releasing 16–20 merozoites which then invade nearby erythrocytes initiating new cycle of invasion. The stage specific effect of carbohybrid **12** was investigated in asexual blood stages. The parasite culture was treated with carbohybrid **12** at different stages i.e. ring, trophozoite and schizont and the effect of treatment was monitored till ring stage of second cycle (56 h). The growth inhibition is maximum when parasite was treated at ring stage as compared to other intraerythrocytic stages. The higher concentration of 15 µM was found to be detrimental to parasite. However, when parasites were treated at schizonts stage, overall percent growth inhibition is only 13.7% even at higher concentration of 15 µM (Fig. 3a). The development of parasite was evaluated by observing Giemsa-stained smears at each stage by light microscopy and the results are presented in Fig. 3b. Initial parasitaemia at ring stage (12–14 h) was taken around ~ 1% for progression assays. In the untreated control after 24 h, 90% of parasites were trophozoite and about 10% schizonts. The images of mid trophozoite stages in control culture depicted increased cytoplasmic size and deposition of haemozoin pigment within the food vacuole (Fig. 3b). In contrast, carbohybrid **12** treated parasite culture failed to develop properly in trophozoite and became small, shrunken with less maturation of the pigment granule. In addition, cultures with 36 h of incubation post-treatment showed no maturation to schizonts stage with condensed nuclear material within the parasite as compared to healthy punctuated schizonts in control. Pyknotic body formation was quite significant in treated cultures. Overall parasitaemia in culture treated at ring stage decreased to 0.5% as compared to 7.2% in untreated control after one cycle (Fig. 3b, graph). Interestingly, when parasite was treated at trophozoite stages, the total parasitaemia reduced only to 4.7% in comparison to 7.2% in control parasites (Fig. 3b, bottom left). Moreover, the parasites treated at schizonts stages showed no significant changes in overall parasitaemia (Fig. 3b, bottom right) indicating carbohybrid **12** is not affecting growth of parasite in schizonts.

### Anti-microtubule activity of carbohybrid **12** is attributed to its binding to tubulin dimer interface

**Fig. 4:**
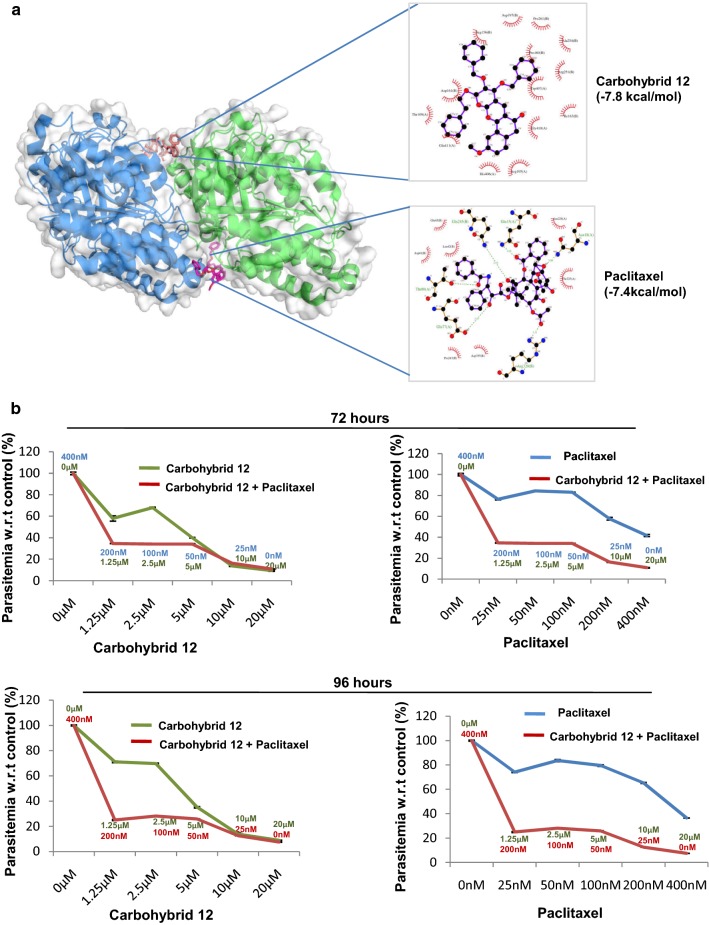
Binding of carbohybrid **12** and paclitaxel to *P. falciparum* tubulin. **a** Figure showing in silico docking of the carbohybrid **12** (brown) to the α,β-heterodimer of tubulin. *P. falciparum* tubulin 3D model depicts dimer of alpha (blue) and beta (green) subunit. Paclitaxel binding site (purple) is present on beta tubulin subunit. **b** Drug combination assay showing the effect of carbohybrid **12** on parasite growth in combination with paclitaxel or both of the drugs alone. Upper and lower panels represent growth patterns of parasites treated with these compounds individually and in combination, for 72 h and for 96 h, respectively. Concentrations of individual drugs used in the combinations are included for each data point. Error bars represent standard error of the mean (n = 2)

Previous reports have demonstrated the effect of anti-malarial drugs via perturbation of microtubules structure of parasite [13]. It has been very well shown that coumarin derivatives exert anti-microtubule activity in cancer cells thereby inhibiting cell division and proliferation of cells [22]. The previously published work reported that the microtubule organization of breast cancer cells was disturbed by treatment with galactal fused pyrano-pyranone derivatives [8]. This led to the hypothesis that *P. falciparum* growth inhibition induced by carbohybrid **12** is accredited to its anti-microtubule activity in the parasite To determine effect of carbohybrid **12** on microtubule dynamics, binding site of carbohybrid **12** on tubulin was characterized by in silico study. Molecular docking of carbohybrid **12** was performed with α–β heterodimer of *P. falciparum* tubulin. Analysis of interacting amino acid residues of tubulin showed strong binding of carbohybrid **12** to interface of α–β heterodimer of tubulin (Fig. 4a). The microtubule stabilizing drug paclitaxel was also tested for structural binding to the heterodimer and it was found to bind to the beta subunit of *P. falciparum* tubulin [13]. The docking studies revealed that carbohybrid **12** binds to tubulin dimer at a different site from paclitaxel. This data is supported by in vitro fixed ratio drug combination assays [23] of carbohybrid **12** with paclitaxel. The effect of all combinations and individual drugs was assessed on the growth of parasite. The overall parasitaemia with respect to untreated control was determined at 72 h (trophozoites) and 96 h (schizonts) in second cycle. Background fluorescence of uninfected erythrocytes was subtracted in order to normalize the readings obtained for growth inhibition assay. The results depict significant reduction in parasitaemia at trophozoite stage, confirming it to be the target stage of carbohybrid. The growth pattern observed for paclitaxel and carbohybrid **12** individually as compared to the combination of these drugs showed that when these two compounds were tested in combination, both of them function at their full potential showing pronounced effect on parasite growth (Fig. 4b).

### Carbohybrid **12** targets the microtubule dynamics of parasite

**Fig. 5:**
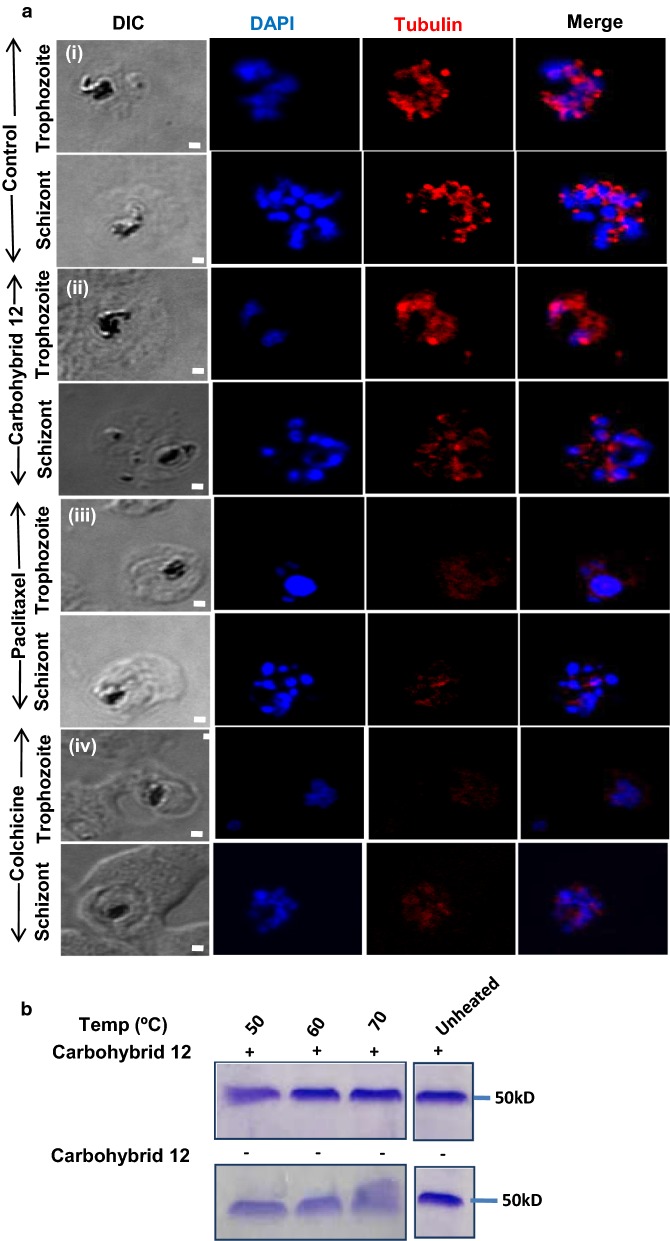
Carbohybrid **12** alters *P. falciparum* microtubules dynamics. **a** Parasites at ring and early trophozoite stages were treated with compounds for 24 h, permeabilized, stained with an anti-α-tubulin antibody followed by Alexa Fluor 546 secondary antibody (red). Nuclear material was stained with DAPI (blue). Panel (i) shows untreated *P. falciparum* erythrocyte. Panel (ii) shows parasite treated with carbohybrid **12** at 6 µM. Panel (iii) shows parasite treated with 500 nM paclitaxel for 24 h. Panel (iv) represent images of parasite treated with colchicine at 10 µM after 24 h. Carbohybrid **12** treated parasites showed diffuse staining of tubulin similar to colchicine. Scale bar = 1 µm. **b** Tubulin protein in presence of carbohybrid **12** shows thermostability at different temperature conditions varying from 50 to 70 ºC

The studies have shown spindle and subpellicular microtubule staining in late blood stages of parasite which gets affected in the presence of drugs [13]. A similar pattern of microtubule staining was observed in untreated culture as parasite progressed through different blood stages of trophozoites and schizonts (Fig. 5a(i)). When treated with 6 µM carbohybrid **12**, the changes were observed in microtubule structure as compared to untreated culture. The punctuate staining and diffused localization of tubulin suggested that carbohybrid **12** destabilizes microtubules (Fig. 5a(ii)). Results were compared with known microtubule stabilizing and destabilizing drugs like paclitaxel and colchicine, respectively. Paclitaxel treatment (500 nM) has shown thick rod-like microtubules in schizont (Fig. 5a(iii)), whereas colchicine treatment (10 µM) resulted in diffused microtubule staining in *P. falciparum* (Fig. 5a(iv)) resembling the staining of carbohybrid **12** treated parasites. This suggests that the microtubule destabilizing effect of carbohybrid **12** is similar to known microtubule destabilizers.

The binding of carbohybrid **12** with tubulin protein was further confirmed by Thermal Shift Assay (TSA). The compound carbohybrid **12** binds to tubulin and provides thermostability to the protein (Fig. 5b).

## Discussion

Targeted therapeutics to diseased cells has beneficial potential in therapies associated with many deadly diseases. The fusion of biomolecules to drug results in better cellular internalization and lower cytotoxicity. Drugs that exhibit limited therapeutic potential, probably due to low bioavailability would show benefit from such approaches. Previous reports showed incorporation of fluoro and sulfonamide moiety to coumarin side chains resulted in better therapeutic properties of synthesized derivative [2, 24]. The antiplasmodial activity of the novel hybrid molecules synthesized by fusion of carbohydrate group to 4-hydroxycoumarin scaffolds has been evaluated to identify potential anti-malarials. The idea behind this type of hybridization is that the presence of carbohydrate group will lead to (i) increased chemical stability of compound. (ii) enhanced interaction of the compound with biological receptors that lead to better uptake. (iii) less toxicity for human cells.

In this study, carbohydrate fused pyrano-pyranones were screened against *P. falciparum* 3D7 strain. As evident from results (Additional file 1: Table S1) and Fig. 1a, galactal fused pyrano-pyranone carbohybrid **12** showed most potent anti-malarial activity among these compounds. The presence of carbohydrate moiety in carbohybrid **12** aids in its better uptake by infected erythrocytes as compared to uninfected erythrocytes. This could be explained by the fact that during asexual stages of life cycle, *Plasmodium* mostly rely on glycolysis for energy production and need constant uptake of glucose via hexose transporters present on parasite [25]. Around 100 fold increase in the glucose uptake is observed in the infected RBC as compared to uninfected RBC [26]. Increased uptake of carbohybrid **12** by infected erythrocytes might be explained by higher expression of hexose transporters on parasite membrane. Further studies in this direction that leads to better understanding of uptake mechanisms in parasite need to be done.

Coumarin derivatives may lead to lysis of erythrocytes by inhibiting different enzymes including glucose-6-phosphate dehydrogenase and carbonic anhydrase [27, 28]. In order to eliminate the possibility of haematolysis, erythrocytes were incubated with different concentrations of carbohybrid **12**, but no significant lysis of erythrocytes was observed even at higher concentration of 50 µM.

Fused pyrano[3,2-c]pyranones derivatives and coumarins, natural or synthetic are used for various pharmacological applications [29, 30]. These compounds target multiple pathways and proteins in a cell thereby inhibit growth and proliferation of cells [1]. Microtubules, which play an important role in cellular motility and cell division, have been recognized as one of the potential anti-malarial drug target [31, 32]. Anti-tubulin agents have been found to be specifically inhibiting the parasite microtubule over the host microtubule [7].

*Plasmodium falciparum* has two alpha and one beta-tubulin gene designated as αI, αII and β, respectively [33–35]. αI and β are transcribed at high rate in both the sexual and asexual blood stages whereas αII is predominantly expressed in male gametocytes, gametes and newly-formed zygotes during sexual blood stages, but later on found to have promiscuous expression in both male and female gametocytes [36]. Tubulin is found to be present in all the stages of asexual erythrocytic cycle. The level of tubulin is found to be increasing from the ring to trophozoite to schizont stages reaching the highest level at the segmenter stage parasite [37]. Previous work with pyrano[3,2-c]pyranones revealed that galactal fused pyrano[3,2-c]pyranones perturbed microtubule organization in breast cancer cells [8]. Molecular docking studies had been carried out to study direct interaction of carbohybrid **12** with *P. falciparum* tubulin. The binding studies indicated different pockets for carbohybrid **12** that lies in the interface of α,β-heterodimer of tubulin to that of paclitaxel. The combination assays describing drug interaction pattern supports this line of evidence. The binding site of carbohybrid **12** distinct from paclitaxel binding site at interface of α,β-heterodimer could explain the pronounced growth inhibitory effect of carbohybrid **12** and paclitaxel combinations. The growth curves for combinations of these two drugs suggest that carbohybrid **12** is more potent anti-malarial in this combination and seems to be mostly responsible for the observed effect. Further validation for the molecular mechanism of carbohybrid **12** anti-malarial activity by immunofluorescence assay indicates that carbohybrid **12** disrupts microtubule dynamics of parasite giving diffuse staining in late stage trophozoites and schizonts after 24 h of treatment in ring and early trophozoite stages. Similar staining patterns were observed in parasite treated with microtubule destabilizing drug, colchicine.

Overall, this study endorses the use of the 4-hydroxycoumarins scaffold with carbohydrate moiety, galactal fusion specifically, for the development of new potent and selective tubulin targeting anti-plasmodial agents.

## Conclusion

These findings encourage novel approaches of carbohydrate group fusion to 4-hydroxycoumarin precursor that resulted in pyrano-pyranones derivatives with better solubility, enhanced uptake and improved selectivity. This study indicates that the galactal fused pyrano-pyranone carbohybrid **12** showed most potent anti-malarial activity against both the chloroquine sensitive (Pf3D7) and chloroquine resistant (PfRKL9) strains with no cytotoxicity. Conjugation to the carbohybrid moiety resulted in enhanced uptake of carbohybrid **12** in the parasitized erythrocytes as compared to its parent compound, 4-hydroxy coumarin. It was shown that the compound acts by destabilizing the microtubule dynamics of the parasite. These data confirms that carbohydrate fused pyrano[3,2-c]pyranones are effective candidates for anti-malarial interventions against *P. falciparum.*

## Supplementary information

**Additional file 1: Table S1.** Antimalarial Screening of compounds. **Figure S1.** Molecular structure of all the compounds (n =30) used in this study. **Figure S2.** Antimalarial effect of galactal fused pyrano-pyranone carbohybrid **12** in chloroquine resistant RKL 9 strain.

## Data Availability

All data generated or analysed during this work are included in the article.

## Abbreviations

JC1: 5,5′6,6′-tetrachloro-1,1′,3,3′ tetraethylbenzimidazolyl carbocyanine iodide
DAPI: 4′,6-diamidino-2-phenylindole
DMSO: dimethyl sulfoxide
AFM: atomic force microscopy
GIA: growth inhibition assay
SAR: structure activity relationship
2-NBDG: 2-[*N*-(7-nitrobenz-2-oxa-1,3-diazol-4-yl) amino]-2-deoxy-d-glucose
∆Ψ_m_: mitochondrial membrane potential
hpi: hours post infection
