# Correction to: Enhanced uptake, high selective and microtubule disrupting activity of carbohydrate fused pyrano-pyranones derived from natural coumarins attributes to its anti-malarial potential

**DOI:** 10.1186/s12936-020-03179-8

**Published:** 2020-03-24

**Authors:** Sonal Gupta, Juveria Khan, Priti Kumari, Chintam Narayana, R. Ayana, Malabika Chakrabarti, Ram Sagar, Shailja Singh

**Affiliations:** 1 Special Centre for Molecular Medicine, Jawaharlal Nehru University, New Delhi, 110067 India; 2 School of Biotechnology, Jawaharlal Nehru University, New Delhi, 110067 India; 3 Department of Chemistry, Shiv Nadar University, NH-91 Dadri, GB Nagar, Greater Noida, UP 201314 India; 4 Department of Life Sciences, School of Natural Sciences, Shiv Nadar University, Greater Noida, India; 5 Department of Chemistry, Institute of Science, Banaras Hindu University, Varanasi, 221005 India

## Correction to: Malar J (2019) 18:346 10.1186/s12936-019-2971-z

Please note, following publication of the original article [1], the authors have advised of two errors that are present in the published article.

Firstly, the two instances of ‘Albumax II’ in the ‘Methods’ section of the article are incorrect: the reagent ‘Albumax I’ should be referred to instead.

Secondly, ‘giemsa’ (also referred to in the ‘Methods’ section) should be capitalized, as ‘Giemsa’.

Finally, an incorrect version of Fig. 4 has been incorporated in the article; please find the correct version of Fig. 4 in this article, for reference.

**Fig. 4:**
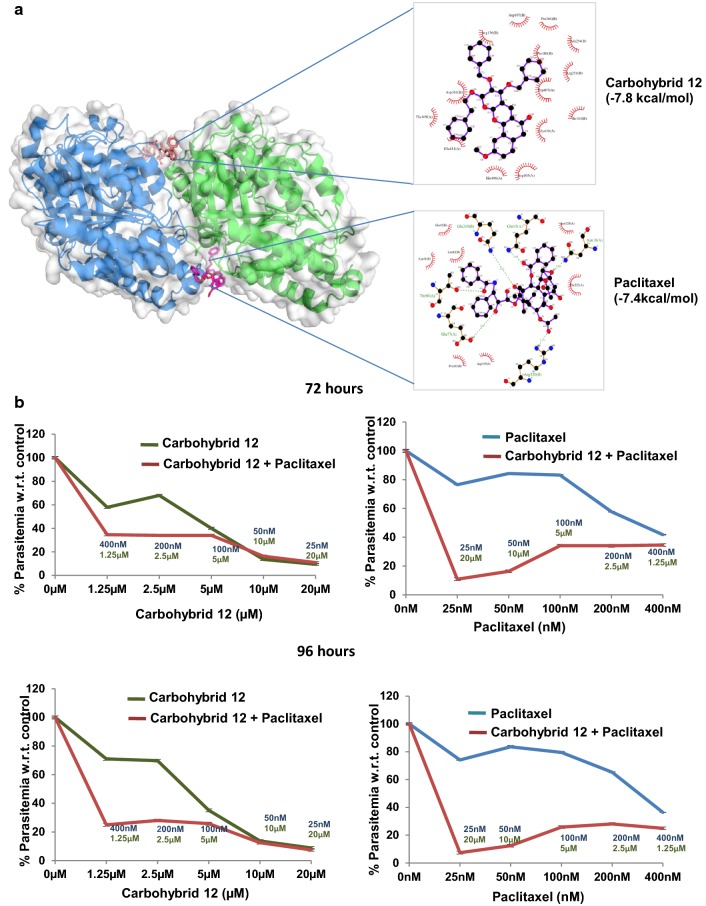
Binding of carbohybrid **12** and paclitaxel to *P. falciparum* tubulin. **a** Figure showing in silico docking of the carbohybrid **12** (brown) to the α,β-heterodimer of tubulin. *P. falciparum* tubulin 3D model depicts dimer of alpha (blue) and beta (green) subunit. Paclitaxel binding site (purple) is present on beta tubulin subunit. **b** Drug combination assay showing the effect of carbohybrid **12** on parasite growth in combination with paclitaxel or both of the drugs alone. Upper and lower panels represent growth patterns of parasites treated with these compounds individually and in combination, for 72 h and for 96 h, respectively. Concentrations of individual drugs used in the combinations are included for each data point. Error bars represent standard error of the mean (n = 2)

The authors apologize for any inconvenience caused.
