# Supplementary material for: Enhanced uptake, high selective and microtubule disrupting activity of carbohydrate fused pyrano-pyranones derived from natural coumarins attributes to its anti-malarial potential
Source: Malar J. 2019 Oct 11;18:346. doi: 10.1186/s12936-019-2971-z (PMC6788091; doi:10.1186/s12936-019-2971-z)
Supplement: Supplementary file 1 — Additional file 1: Table S1. Antimalarial Screening of compounds. Figure S1. Molecular structure of all the compounds (n =30) used in this study. Figure S2. Antimalarial effect of galactal fused pyrano-pyranone carbohybrid 12 in chloroquine resistant RKL 9 strain. [file 12936_2019_2971_MOESM1_ESM.docx]

**Enhanced uptake, high selective and microtubule disrupting activity of carbohydrate fused pyrano-pyranones derived from natural coumarins attributes to its antimalarial potential**

Sonal Gupta^a†^, Juveria Khan^b†^, Priti Kumari^c†^, Chintam Narayana^c^, R. Ayana ^d^, Malabika Chakrabarti^a^, Ram Sagar^e^* and Shailja Singh^a^*

*^a^Special Centre for Molecular Medicine, Jawaharlal Nehru University, New Delhi-110067,India*

*^b^School of Biotechnology, Jawaharlal Nehru University, New Delhi-110067,India*

*^c^Department of Chemistry, Shiv Nadar University, NH-91 Dadri, GB Nagar (UP) 201314, India*

*^d^Department of Life Sciences, School of Natural Sciences, Shiv Nadar University, Greater Noida, India.*

*^e^Department of Chemistry, Institute of Science, Banaras Hindu University,Varanasi, 221005,India,*

*Corresponding Author:

*Email:*[shailja.jnu@gmail.com](mailto:shailja.jnu@gmail.com), [ram.sagar@bhu.ac.in](mailto:ram.sagar@bhu.ac.in)

**^†^**equal contribution

| **Compound** | ***P. falciparum* (10µM)** | ***Hep G2* (100µM)^a^** |
| --- | --- | --- |
| 1 | -8.3±0.35 | -29.4±0.04 |
| 2 | -0.50±0.33 | -01.1±0.08 |
| 3 | -12.36±1.25 | -15.9±0.02 |
| 4 | 0.018±2.26 | -13.9 ±0.10 |
| 5 | -6.19±0.70 | -45.5 ±0.11 |
| 6 | -12.95±0.76 | -24.4±0.10 |
| 7 | -14.05±0.49 | 10.6±0.05 |
| 8 | -13.56±0.63 | 01.7±0.43 |
| 9 | -13.76±0.54 | -05.7±0.25 |
| 10 | -6.22±0.46 | 08.0±0.22 |
| 11 | 15.57±0.43 | -12.9±0.26 |
| 12 | 47.56±0.42 | -18.0±0.07 |
| 13 | -8.86±0.14 | -8.2±0.06 |
| 14 | 31.41±0.13 | -14.9±0.08 |
| 15 | 2.82±0.31 | 01.2 ±0.48 |
| 16 | -5.77±2.39 | 04.0±0.17 |
| 17 | 53.28±0.32 | 07.6±0.01 |
| 18 | -1.95±0.18 | -15.2±0.23 |
| 19 | -3.55±0.96 | -04.4±0.22 |
| 20 | 32.38±0.62 | 04.8±0.51 |
| 21a/b | 2.37±0.95 | -19. ±10.05 |
| 22a/b | 15.68±0.29 | 19.0±0.37 |
| 23a/b | -5.6±0.73 | 06.3 ±0.06 |
| 24a/b | 2.26±0.45 | 13.9 ±0.22 |
| 25a/b | 1.02±0.39 | -02.8±0.37 |
| 26a/b | -9.95±0.72 | -03.7 ±0.14 |
| 27a/b | 38.57±0.95 | 02.5±0.45 |
| 28a/b | -4.28±0.65 | 06.4±0.03 |
| 29a/b | 28.76±0.78 | -00.4±0.10 |
| 30a/b | -2.29±1.0 | -08.3±0.11 |

**Table S1. Antimalarial Screening of compounds**

**Growth Inhibition (%) ± S.D.**

**^a^** work on HepG2 previously published in ( Kumari P *et. al.* 2018)

**Figure S1.** Molecular structure of all the compounds (n =30) used in this study.

**Figure S2.** Antimalarial effect of galactal fused pyrano-pyranone carbohybrid **12** in chloroquine resistant RKL 9 strain.
